# Can Aromatherapy Make People Feel Better Throughout Exercise?

**DOI:** 10.3390/ijerph17124559

**Published:** 2020-06-24

**Authors:** Sungho Kwon, Jihoon Ahn, Hyunsoo Jeon

**Affiliations:** 1 Department of Physical Education, Seoul National University, Seoul 08826, Korea; sh1kwon@snu.ac.kr; 2 Department of Physical Education, Chosun University, Gwang-ju 61452, Korea; lmjhs1124@chosun.ac.kr

**Keywords:** aromatherapy, exercise, feelings, positive psychological effects, exercise satisfaction

## Abstract

We analyzed participants’ feelings and arousal before, during, and after exercise as per whether they receive aromatherapy. Twenty university students who regularly took part in health exercises were selected through purposive sampling. Changes in feelings were measured through a 2D circumplex model and an in-depth interview. The effects on exercisers who received aromatherapy were more positive than for those who did not receive any treatment. Specifically, it induced positive feelings during exercise, reduced fatigue during exercise, and improved participants’ feelings during the recovery period. Aroma has a key influence on exercisers’ feelings, and it can positively influence exercise satisfaction and persistence.

## 1. Introduction 

Exercise psychology researchers have proposed that exercise increases vitality and positively affects various psychological factors such as anxiety, depression, self-esteem, mood, feelings, etc., [1,2]. However, most studies that address the effects associated with exercise have only compared participants’ feelings before and after exercise; the psychological reactions that appear during exercise have not been systematically verified. Because most studies have analyzed the overall average of exercisers, there is a lack of information on individual differences [3]. Backhouse et al. [3] emphasized that to understand the effects that are experienced during exercise in detail, changes must be examined before, during, and after exercise and the effects experienced by participants must be analyzed not at an aggregate level, but by individuals or small groups with similar characteristics.

Moreover, there is a need to analyze the reasons behind why over 50% of participants quit exercising after approximately 6 months despite the psychological benefits [3]. The reasons may be attributable to various personal factors, social factors, exercise programs, and so on; however, the feelings or mood that is experienced during exercise may be a prominent issue [4]. In general, an individual’s feelings are closely related to one’s sense of happiness, how they address stressful situations, and quality of life [5,6].

The feeling or mood that is experienced by an individual while exercising can occur uniquely as per the person’s psychological state; however, it generally differs by the senses that are recognized while exercising [7,8]. When an individual exercises in a friendly environment, their physical senses generate a positive energy which encourages the exerciser’s motivation and adherence [9].

Because a person’s physical and psychological state are inseparable, the five senses are used to guide one’s mind [10,11]. Our senses play an important role in receiving information from our environment, and these senses deliver information to the brain that helps us heal [12]. Despite the many studies on the five senses, sense of smell is the least evaluated [13] because olfactory studies require greater stimulus control accuracy and there are experimentation difficulties [14]. But, studies on whether feelings change as per sense of smell during exercise will be important for health researchers. Because our sense of smell continues to work as we exercise, there is need for a detailed experimental study regarding how smell affects feeling and arousal. 

Smells influence an individual’s cognitive ability, attention, memory, and sleep, and are a representative medium that directly interacts with a person’s emotions [15,16]. This fact was made known in the early 1920s when Gatti and Cayola found that inhaling essential oils has soothing and stimulating effects [17]. Aromas are widely used in clinical studies because they are easy to use, can be used regardless of time and place, act quickly, and have almost no side effects [18].

Aromatherapy uses the characteristics of aromatic oils extracted from various natural plants to maintain or promote health [19]. When the particles are inhaled, most of the particles enter the lungs and the rest are delivered to the brain. All the particles that are sent to the brain are delivered to the brain cortex and limbic system from the *sulcus olfactorius*; therefore, an emotional reaction occurs in addition to the delivery of actual information [20]. Such reactions secrete substances such as peptides like endorphins through functions that activate parasympathetic nerves and calm excited sympathetic nerves. They induce a state of happiness accompanied with calming and tension relaxation effects and suppress anxiety, rage, fear, etc., [21].

Aromatherapy is used in diverse ways in sports. For example, inhaling peppermint essential oil was found to improve grip, high jump records, long jump records, pulmonary function [22], calm the bronchial smooth muscle, and increase the concentration and consumption of oxygen to the brain [23]. Another study found that speed and push-up count had increased significantly after having 40 adults smell peppermint during exercise [24], while another found that momentum and heart rate recovery had improved after inhaling aromatic fragrances [25]; therefore, it appears clear that aromatherapy improves bodily functions and positively affects athletic performance.

Unfortunately, most studies related to aromatherapy have had results which are more focused on the physiological effects of aromatic fragrances rather than their psychological effects. However, aromatherapy has many psychological effects as physiological effects, and it shows the most influence on subjective stress and mood [26]. It was found that the inhalation of essential oils provides a cost-effectiveness, safeness, and appropriate therapy to someone with a mental illness [27]. Human studies with inhalation of aroma essential oil have shown that the inhalation reduced symptom of anxiety and depression [28], and enhanced mood and relief [29]. Since aromatherapy’s psycho-physiological effects reducing physical pain and improving mood have been proven, applying aromatherapy in healthy exercise participants can be a good alternative way to avoid feeling bad during a workout.

Therefore, present study tried to examine the effect of aromatherapy on exercise participants’ feelings before, during, and after exercise. Consequently, we compared and analyzed participants’ feelings and arousal before, during, and after exercise as per whether they receive aromatherapy; then, we examined the psychological effects of aromatherapy on exercise based on these results.

## 2. Methods

### 2.1. Participants

Twenty university students (10 men, 10 women, M_age_ = 22.5 years) who regularly participate in aerobic exercises such as jogging and biking (approximately 2 times a week) were selected through purposive sampling, which is a type of nonprobability sampling. Participants had no olfactory disabilities.

### 2.2. Measures

After setting an appropriate heart rate for each research participant (70–80% of their maximum heart rate) through maximal exercise testing, a test on change in feelings and physical arousal was conducted when aromatherapy was received and when aromatherapy was not received while running on a treadmill for 30 min at a fixed setting. In addition, the feelings and activation as per time were presented through a 2D circumplex model, which was used as a basis for examining the participants’ feelings during exercise in detail through an in-depth interview.

### 2.3. Maximal Exercise Testing

A heart rate monitor (Polar S610, Polar Electro, Kempele, Finland) was used to measure maximal heart rate. Physical health instructors measured the testing and conducted the experiment as per exercise participants’ perceived exertion. Maximal exercise testing was conducted on a treadmill (Quark CPET, Cosmed, Rome, Italy) based on the Bruce protocol (i.e., an exercise tolerance test). The starting point (stage 1) was 1.7 mph at a 10% grade for 3 min, followed by stages (3 min each) with incremental increases in both grade (by 2%) and speed (by 0.8–0.9 mph). The test was performed until the participants became exhausted and the highest heart rate at the end of the test was set to the participant’s maximum heart rate.

### 2.4. Psychological Testing (Pre-, Intra-, and Post-Exercise Valence and Activation)

A two-dimensional circumplex model was used [3,30], which comprises two dimensions of activation (high arousal, low arousal) and valence (pleasant, unpleasant). Affective valence and activation were assessed using the 11-point (from –5 (*I feel very bad)* to +5 (*I feel very good*) Feeling Scale (FS; [31]) and a modified 6-point version, from 1 (*low arousal*) to 6 (*high arousal*), of the Felt Arousal Scale (FAS; [32]). Both the FS and FAS have been utilized in past exercise research conducted by many experiments around the world that have provided satisfactory validity [3].

### 2.5. Aromatherapy Method 

Aromatherapy can be performed several ways (e.g., massage, skin application, inhalation, foot bath, aromas, etc.,). This study used inhalation, which is considered the most effective method. The lamp diffusion method was also used as it is the most common and easiest among inhalation methods. The effects of the lamp diffusion method can be seen directly, and because its effects are quick, it is often used to calm psychological tension or inspire moods and feelings [33]. As the fragrance begins to diffuse through the lamp, everyone in the room can receive the effects of aromatherapy. A specialist was consulted to select one of over 40 different aromatic oils that were suitable for the experiment. Based on the specialist’s opinion, we selected an orange fragrance from the citrus class, which is popular and familiar to both men and women and has effects that improve mood [34].

### 2.6. In-Depth Interview 

To examine the feelings that change before, during, and after exercise in more detail as per aromatherapy, there was an in-depth interview with each research participant 10 min after each experiment. The interview that took place after the first experiment included questions about the participant’s physical and psychological remarks before the experiment, their feelings toward the indoor environment, and their feelings from the beginning of the exercise up to 10 min after the end of the exercise. The interview that took place after the second experiment included questions regarding differences in the participants’ body or feelings from the first experiment and additional questions that asked if the participants were aware of the fragrance and about the effects of the aroma. The interviews were conducted face-to-face with each individual for approximately 15–20 min, and they were recorded with participants’ consent. To help the participants remember the feelings and physical arousal they experienced while exercising in detail, they were shown a 2D circumplex model pertaining to before, during, and after exercising.

## 3. Procedure

The research participants ran on the treadmill for 30 min twice. Aromatherapy was only performed during the second experiment when participants exercised. The participants completed the first experiment; then, the same participants completed the second experiment one week later (on the same day of week). In the second experiment, through the aroma lamp installed in the laboratory, participants carried out the exercise by inhaling aroma from the beginning to the end. Furthermore, because prior knowledge of the relationship between aromatherapy and feelings can affect the results of the study, the participants did not know that they would be experiencing aromatherapy. The experiments were conducted in an indoor area that was a suitable size for aromatherapy.

Exercise intensity was controlled by setting it to each participant’s target heart rate (70–80% of their maximum heart rate). The research assistants consistently checked the exercisers’ heart rate during exercise and adjusted the speed of the treadmill accordingly. Their feelings and physical arousal were measured right before exercising, every 5 min during exercise, and 10 min after exercise. A 2D circumplex model was used to examine the change in feelings and physical arousal before, during, and after exercise for the first and second experiment, and the 2D circumplex model was divided into activation (high/low arousal level) and valence (comfortable/uncomfortable). 

### 3.1. Data Analysis

To analyze feelings that changed during exercise, we showed the status of the research participants by their responses as per the flow of time (5-min intervals) through a 2D circumflex model. The recorded data and memos acquired through in-depth interviews were transcribed. When the recorded data were transcribed, the same expressions used by the participants were used whenever possible so that the meaning that the respondent intended to deliver would stay intact. The transcription was conducted by the investigator so that the meaning of the data would be reflected in the study results as is. An analytic memo was written for each participant through the transcribed interview data. All responses regarding each semi-structured question were coded in-depth and the core meaning implied in the data was summarized. The collected data were categorized using specific factors so that they could be organized systematically.

### 3.2. Ethical Considerations

This study, including participant recruitment, research procedures and methods to resolve ethical problems, was reviewed and approved by the Institutional Review Board at Seoul National University (IRB No. 1802/001-002). The study was only conducted after obtaining such IRB approvals.

## 4. Results

### 4.1. Calculating Target Heart Rate through Maximal Exercise Testing

Maximal exercise testing was conducted before beginning the experiment to calculate the maximum heart rate and target heart rate for each participant (Table 1).

### 4.2. Physical and Feeling Changes before, during, and after Exercise as per Aromatherapy

Physical arousal and feelings were measured before exercising, every 5 min while exercising, and 10 min after exercising. Then, an in-depth interview was conducted.

**Figure 1:**
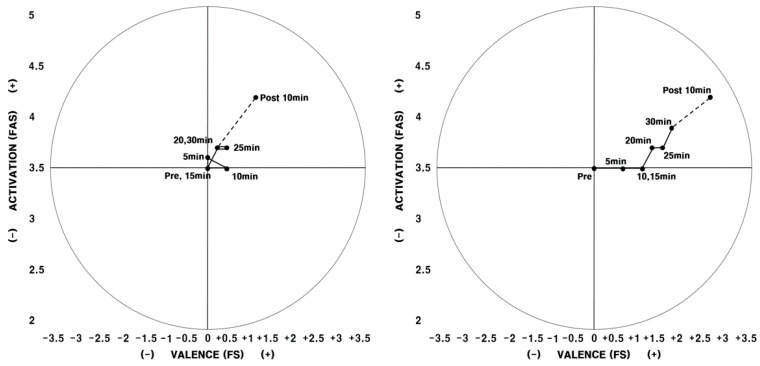
2D circumplex model (average FAS and FS of all participants) Note. Left: 1st experiment/Right: 2nd experiment. FAS: felt arousal scale; FS: feeling scale.

*Average of all research participants as per aromatherapy.*Figure 1 shows the results of the average FAS and FS that were measured as research participants exercised.

There were small continual changes in physical arousal and feelings as research participants ran on the treadmill during the first experiment; however, these results were generally distributed closely around the reference point. There were slight positive changes in the physical arousal and feelings that were measured 10 min after exercising. In other words, physical arousal and feelings repeatedly showed slight increases and decreases after beginning exercise; then, they increased in a positive direction once the participant completed the exercise and rested.

In the circumflex model for the second experiment, when aromatherapy was conducted, physical arousal and feelings that were perceived during exercise increased as time passed. Compared to the first experiment, there was no major difference in physical arousal; however, feelings significantly improved. Unlike in the first experiment, where no changes in feelings were seen during exercise, feelings continuously improved during exercise in the second experiment and were maintained after exercising. Figure 1 shows research participants’ average physical arousal and feelings as per exercise time, and the circumplex models for everyone varied with different patterns. Based on the argument made by Backhouse et al. [3], that feelings must be examined on an individual level or by small groups rather than on an aggregate level, the participants were categorized into four groups based on their individual 2D circumplex models and in-depth interviews.

**Figure 2:**
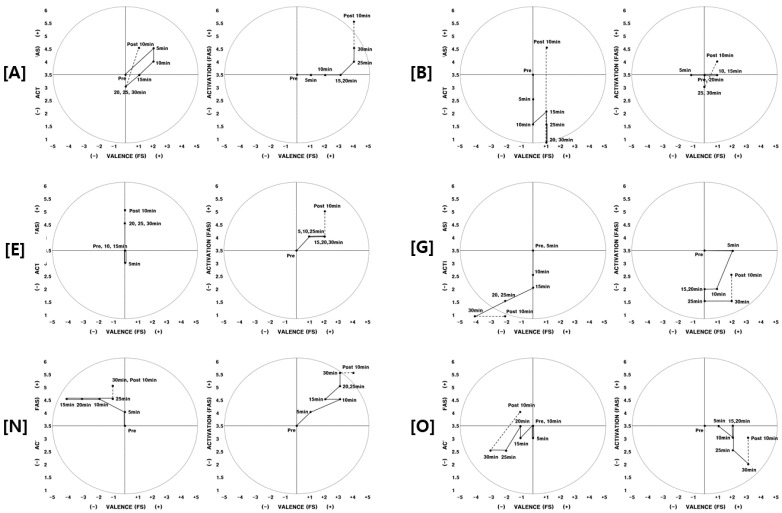
2D circumplex model (participants A, B, E, G, N, O) Note. Left: 1st experiment/Right: 2nd experiment. FAS: felt arousal scale; FS: feeling scale.

Participants who were aware of the fragrance and experienced positive feelings while exercising are (A, B, C, E, G, H, J, K, N, O, P, R). Figure 2 shows the patterns of change in physical arousal and feelings that were measured while participants (A, B, E, G, N, O) exercised.

During the second experiment, when aromatherapy was conducted, the scores of feelings had increased. There were larger range of increase for feelings than for physical arousal, and feelings continuously increased from the beginning of the exercise until the participant rested. Participant A was tired before the second experiment; however, she felt physically and emotionally refreshed once she began exercising. She said that the fragrance affected her fatigue and feelings while exercising.

*“Before exercising, I was so worried because my body felt so heavy. But, after about 15 minutes, my mood started to improve. There was a fresh fruit smell or a lemon tea smell; so, I was able to exercise in a much more refreshing environment. What was interesting is that I didn’t feel like the intensity of the exercise was higher than before.” *

(Participant A)

Regarding the differences between the two experiments after they ended, Participant A and B said that the fragrance influenced what she felt while exercising. They also answered that there was difference in the degree of fatigue she felt from the same exercise intensity.

*“The room temperature and other conditions were similar; but, because of the smell, I felt like it was more fresh and comfortable. The exercise was at the same level of intensity; but, it was interesting because it felt less difficult.” *

(Participant A)

*“I had never used aromas before; so, I didn’t know anything about the effects of aromas. But I think I was in a better mood because I was exercising while smelling that fragrance.”*

(Participant B)

Participant E also had comparable results (Figure 2). There was no change in feelings during the first experiment, and physical arousal had increased as time passed. However, feelings had changed positively while exercising during the second experiment, and she said that exercising was not as difficult as it was in the first experiment because of the refreshing smell. Participant E ran at the same speed and heart rate on the treadmill for both the first and second experiment; however, she said it was less difficult during the second experiment. Although she participated without knowing about the aromatherapy, she responded that the pleasant smell had an effect.

*“I was running at a similar speed and running to match the same heart rate as last time; but, it wasn’t as hard as I expected. I think I kept running on the treadmill while wondering why that was. I think starting off in a good mood because of the pleasant smell could be the reason.” *

(Participant E)

Participant G’s physical arousal decreased as time passed during the first experiment, and their feelings worsened starting from 15 min into the exercise until the exercise ended. During the second experiment, physical arousal also decreased as time passed. Feeling had repeatedly increased and decreased (Figure 2); however, it was generally positive compared to the first experiment. He picked “fragrance” to be the biggest factor as to why the results of the two experiments differed.

*“The difference in today’s experiment (second experiment) was that there was a sweet orange smell. I don’t remember anything about the smell from last time; but, I definitely remember it this time. It felt refreshing while smelling orange smell as I was running.”*

(Participant G)

Participant N and O, who experienced opposing feelings from the first and second experiment, they explained that since there was a pleasant smell while exercising, their feelings kept improving from the start of the exercise (Figure 2). Compared to the first experiment, participant N’s feeling measured 10 min after exercising had also increased. Since they were exercising in a good mood, they felt like the time of the experiment was shorter and their fatigue while exercising decreased.

*“I usually feel in a better mood after exercising; but, my body still feels light even after I ran at a relatively fast speed today. Since I ended the exercise in a good mood, I think that mood lasted and I feel proud of myself.” *

(Participant N)

*“Since I kept smelling that pleasant smell, I think I concentrated more on the smell than on exercising. I kept trying to figure out where it was coming from. That’s why I think the time went faster, and it felt much less difficult than last time.” *

(Participant O)

**Figure 3:**
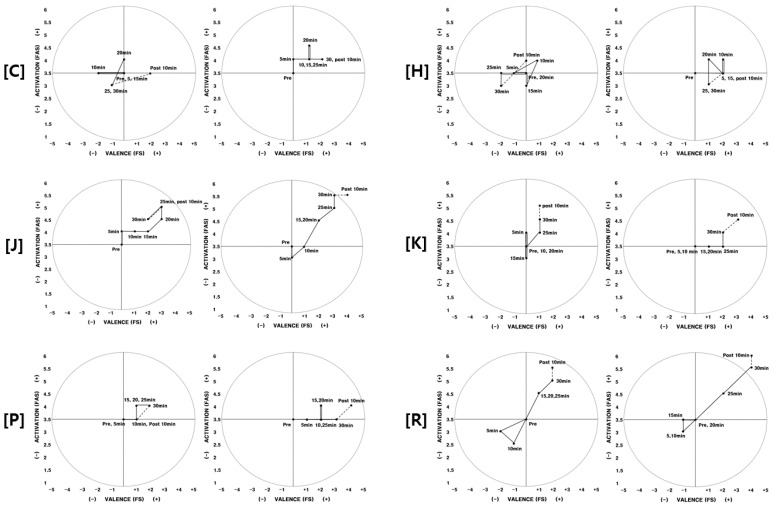
2D circumplex model (participants C, H, J, K, P, R) Note. Left: 1st experiment/Right: 2nd experiment. FAS: felt arousal scale; FS: feeling scale.

In addition to the above participants, eight other participants were found to have experienced a positive psychological influence from the aromatherapy. They said they did not have any psychological or physical concerns that would be relevant to the results of this study before the first and second experiment. Data from in-depth interviews with similar content were omitted. The 2D circumplex models of these participants (C, H, J, K, P, R) are shown in Figure 3.

**Figure 4:**
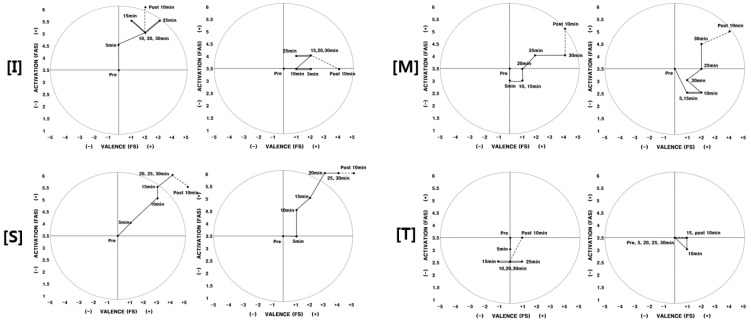
2D circumplex model (participants I, M, S, T) Note. Left: 1st experiment/Right: 2nd experiment. FAS: felt arousal scale; FS: feeling scale.

*Participants who were aware of the fragrance, but did not experience positive effects were (I, M, S, T).* Participants I, M, S, and T were aware of the fragrance during the second experiment; however, no differences were found, and they did not experience a positive influence on their physical arousal or feelings (Figure 4).

Participant I experienced lower feeling and physical arousal while exercising during the second experiment than the first experiment. Participant M’s feeling gradually changed positively as time passed while exercising during the first experiment; however, her feeling improved, then grew worse, during the second experiment. Her physical arousal decreased after she started exercising for both experiments, then improved after 10–15 min had passed. However, the range of increase in physical activation while exercising had changed for the second experiment. However, as with Participant I, her feeling during the time interval from the end of the exercise (after 30 min) until 10 min afterwards had increased in the second experiment, which did not occur during the first experiment. During the in-depth interview, Participants I and M responded that they were unable to feel the effects of aromatherapy because of their poor physical condition, and they explained that there were other factors that affected their physical arousal and feeling.

*“To be honest, I had to work in the morning; so, I took part in the experiment while being short on sleep. That’s why my body felt heavier than during the first experiment. I don’t think I was in a good mood.” *

(Participant I)

*“The sweet smell lasted from the beginning to the end of the exercise. I think the smell was good enough to improve my mood. But, I played soccer for a long time yesterday; so, my legs hurt a lot.”*

(Participant M)

Participant S had the similar circumplex model patterns for both the first and second experiment. Both physical arousal and feelings had continuously increased after starting to exercise for both experiments. For Participant T, the range of change in physical arousal and feelings was negligible compared to other participants. Aside from how physical arousal decreased slightly during the first experiment, there was no difference in the second experiment. Based on the in-depth interviews with Participants S and T, they did not experience a difference in feelings or physical arousal between the first and second experiment, and they were not able to see positive effects from the fragrance that they perceived.

*“I’m usually in a good mood when I’m exercising. Even today, I felt my body getting lighter and my mood improving as time passed.” *

(Participant S)

*“I did think there was a good smell; but, I don’t think there was a particular difference because of the smell because it’s not like there was a bad smell last time.” *

(Participant T)

**Figure 5:**
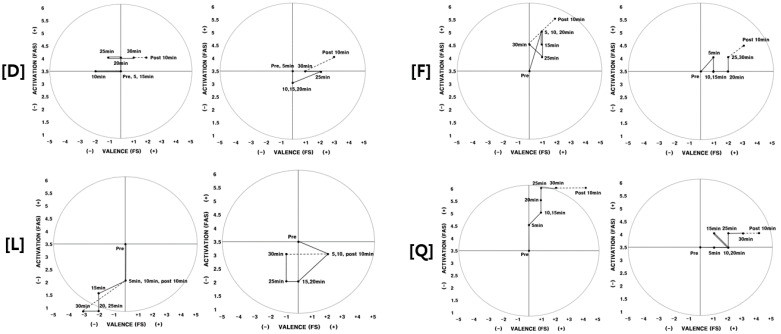
2D circumplex model (participants D, F, L, Q) Note. Left: 1st experiment/Right: 2nd experiment. FAS: felt arousal scale; FS: feeling scale.

Participants who were not aware of the fragrance but experienced a positive change in feelings during the second experiment were (D, F, L). Participants D, F, and L were not aware of any smell in the experiment area until the end of the experiment. They were unable to experience any differences in their exercise environment for both experiments; however, they did experience a difference in their physical/psychological state (Figure 5). 

Participant D experienced negative feelings while exercising during the first experiment; however, she did not experience negative feelings during the second experiment. Participant F and L had also exercised with better feelings during the second experiment than the first experiment. Based on the in-depth interviews after the experiment, they were not aware of the aroma; however, they revealed that they were more comfortable during the second experiment.

*“I didn’t see a particular difference in the experiment settings compared to the last experiment. My condition was similar, too. One difference I felt while running this time was that it was less difficult even though I was running to match the same heart rate as last time.” *

(Participant D)

*“Thirty minutes didn’t feel like such a long time. But, I didn’t feel any particular difference in the environment; I just thought my body was in good condition today.” *

(Participant F)

*“I was having a really hard time by the end of the first experiment. But, during today’s experiment, I had a less difficult time as time passed while exercising.” *

(Participant L)

The exercisers in this category also said they did not have any psychological or physical concerns that would be relevant to the results of this study before the first and second experiment.

*Participants who were not aware of the aroma and did not experiment positive effects (Q).* Participant Q’s physical arousal increased as time passed during the first experiment. He felt his body grow lighter and his physical arousal level increased. However, there was almost no change in physical arousal during the second experiment (Figure 5). His feeling before, during, and after exercising was generally positive for both experiments.

*“Since I wasn’t told about the fragrance before the experiment, I wasn’t aware of any smell. I felt like my body was lighter during the first experiment. Since I didn’t smell anything, I don’t think my mood improved while exercising today (second experiment) because of the fragrance.”*

(Participant Q)

### 4.3. Positive Effects of Aromatherapy that Appeared before, during, and after Exercising

Common effects that were observed from the participants through their 2D circumplex models and in-depth interviews were compiled together. The effects of aromatherapy were divided into three major categories. The fragrance induced positive emotions while exercising and thereby increased feelings and enabled participants to maintain those positive feelings even after exercising. It made exercisers feel like the intensity of the exercise was lower, which in turn lowered their fatigue while exercising. Table 2 shows the detailed differences between the two experiments.

## 5. Discussion

Feeling or mood, which is an important variable that can determine whether an individual continues or quits exercising [4], is highly influenced by various senses that are perceived while exercising [7]. If the exercise environment is comfortable, people’s physical and psychological state will be positive, maximizing the effects of exercising and enabling them to continue exercising [9].

Although sense of smell is a medium that directly interacts with our emotions [15], studies regarding this sense are lacking compared to other senses [14]. The effects of aromatherapy, which is known to improve health [19], have only been verified on a physiological basis, and there are no studies that have closely examined sense of smell and psychological state during exercise.

Therefore, we compared and analyzed the feelings and physical arousal of healthy exercisers as per whether they received aromatherapy. We examined the psychological effects of aromatherapy while exercising. To understand the feelings that are experienced while exercising in detail, the changes in feelings were examined before, during, and after exercise, both at an aggregate level and an individual level to supplement existing studies’ limitations.

Regarding the feelings and physical arousal of all exercisers, upon examining the study results presented through 2D circumplex models, there was no major change in participants’ feelings while exercising during the first experiment. However, feelings increased with the passing of time during the second experiment. While a detailed analysis is difficult because the differences appeared as an average of all participants, participants’ feelings during the second experiment with aromatherapy was much more positive and effective.

To understand the differences between the first and second experiment in more detail, each participant’s in-depth interview data and circumplex models were analyzed to explain the detailed effects of aromatherapy. First, because the degree of feelings and slope of change differed for each participant, we were unable to standardize the results into one pattern. This supports the argument made by Backhouse et al. [3] that an analysis on feelings must be handled at an individual level. Although some participants supported the results of preceding studies that found that exercisers’ feelings worsened as time passed and the amount of exercise increased [3,35,36], there were also cases where participants experienced almost no change in feelings while exercising or experienced positive changes. This implies that there are distinct factors that influence an individual’s feelings, and that the individual’s subjective standards are reflected regarding the suppression of feelings. In this study, we tried to control the physical and emotional conditions of the participants as much as possible by measuring the feelings and physical arousal at the beginning of the experiment. However, it is important to recognize that the studies related to personal emotion and feelings should be conducted at a personal level and the interpretation of the results can become too diverse, because the individual’s personality and sensitivity to smell can affect the feelings during the experiment. 

Numerous studies have examined the psychological influence of vision and sound on participants while they exercise [8,37,38,39]. For example, when a person warms up, works out, and cools down with music they like, it helps them psychologically [40]. Studies on whether feelings change as per sense of smell during exercise will also be important for health researchers. Because our sense of smell continues to work as we exercise, there is need for a detailed experimental study regarding how smell affects feeling and arousal.

Many participants experienced an increase in positive feelings during the second experiment compared to the first experiment, implying that the aroma had a positive impact. The in-depth interviews supported this and showed that induced positive feelings reduced the subjective intensity of exercise, reduced fatigue while exercising, and improved feelings after exercising. This supports previous results that fragrances have a positive influence on people’s subjective stress and feelings [26] and increases the amount people exercise [22,24].

Based on the in-depth interviews, participants’ responses were categorized into three major categories to explain the effects of aromatherapy. First, exercising induced positive feelings. Participants experienced comfort and freshness while exercising in the environment where aromatherapy was conducted, and they reported a low level of anxiety regarding exercising. The psychological effects of aromatherapy on patients with various diseases (e.g., insomnia, dementia, etc.) are well-known [20,41]. While there are slight differences as per type of fragrance, aromatherapy generally decreases anxiety and emotional stress and increases feelings [42,43]. Some people also use aromas to control their emotions or maintain mental stability [44]. Therefore, simply being exposed to fragrances can positively influence feelings.

Second, fatigue decreased while exercising with aromatherapy. Although the participants exercised for the same amount of time and intensity, they felt that exercising was less difficult during the second experiment and reported that the time went faster. The fact that participants who exercised in a physically aroused state during both experiments felt less physical burden during the second experiment can be interpreted as a positive effect of aromatherapy. This was consistent with Sakamoto et al. [45]. Because the distortion of exercise time is one of the most representative phenomena of the flow concept proposed by Csikszentmihalyi [46], future studies should address this connection.

Lastly, after exercising, participants showed improved feelings. This is a prominent issue in sports science and has been verified through many studies [47]. In other words, when a person’s feelings improve through exercising, they will try to continue exercising. In this study, participants’ feelings, which was measured 10 min after exercising, was higher during the second experiment with aromatherapy than during the first experiment, implying that the effect of the aroma was related to continued exercise. This is consistent with Grimes [48], who found that perceiving fragrances positively affected attachment and persistence.

This study had some limitations. A small number of participants were not aware of the aroma; however, they still experienced positive feelings during the second experiment, even though the environment was the same in both experiments (apart from the aromatherapy). Because the knowledge of the participants in the relationship between aromatherapy and feelings can influence self-perception of improvement, therefore, there is a need to confirm whether the effect of aromatherapy occurs regardless of whether the exerciser is aware of the smell. In one case, a participant exercised while being physically tired, and reported negative feelings during the second experiment even though he/she was aware of the aroma. Follow-up studies should consider that the effects of aromatherapy may not appear when the participant’s physical or mental condition is extremely low. In the same vein, since the effects of aromatherapy may vary depending on the personal characteristics of an individual, future studies are also needed to explain the relationship between feelings resulting from aromatherapy and personal characteristics. There is also a need to diversify the experimental design to explain the influence of aromas on psychological state during exercise in more detail. For example, studies should control for participants’ age, frequency of exercise, exercise intensity, and use various aromatherapy methods and fragrances.

## 6. Conclusions

This novel study verified the effects of sense of smell on feelings while exercising, while previous studies had only addressed sense of smell and physiological effects. We showed that olfactory aspects must be considered in the exercise environment alongside visual and auditory factors. Overall, these results are useful for policymakers in the public health field, and they can be used to improve feelings and persistence when implementing exercise programs at sports facilities.

## Figures and Tables

**Table 1:** Maximum heart rate and target heart rate.

Participant	HR Max	Target HR	Participant	HR Max	Target HR
		70%–80%			70%–80%
A	198	138–158	K	193	135–155
B	199	139–159	L	202	142–162
C	202	142–162	M	199	139–159
D	201	141–161	N	193	135–155
E	199	139–159	O	202	142–162
F	201	141–161	P	202	142–162
G	198	138–158	Q	198	138–158
H	202	142–162	R	202	142–162
I	201	141–161	S	202	142–162
J	196	137–157	T	200	140–160

Note: HR: heart rate.

**Table 2:** Positive effects of aromatherapy as described by participants.

Effect	Detailed Experience
It induced positive emotions while exercising	I felt fresh and refreshed while exercising.
	I felt like it was a comfortable environment and felt mentally comfortable.
	I was in a good mood because the smell was pleasant and familiar.
	My anxiety regarding the difficulty of the exercise decreased.
	The smell persisted; therefore, I could keep exercising in a good mood.
	I felt like exercising was less difficult, which made exercising more fun.
Decreased fatigue while exercising	I felt like I was in good physical condition.
	Exercise time felt short.
	Physical fatigue was less than during the 1st experiment, even though I was running at the same speed and matching the same heart rate.
	My legs hurt less while running than they did during the 1st experiment.
	I ran until I was physically aroused; however, I did not feel that it was difficult.
	Even though time passed, the level of difficulty was the same as in the beginning.
Improved feelings after exercising	My mood improved after exercising and I felt like I could exercise more.
	Instead of becoming drowsy after exercising, I felt like my body was light.
	My improved feelings while exercising persisted.
